# Antiaging Factor Klotho Retards the Progress of Intervertebral Disc Degeneration through the Toll-Like Receptor 4-NF-*κ*B Pathway

**DOI:** 10.1155/2020/8319516

**Published:** 2020-03-19

**Authors:** Fangfang Bi, Wenbo Liu, Zongtao Wu, Chen Ji, Cuicui Chang

**Affiliations:** ^1^Department of Medicine, Xi'an Peihua University, Xi'an 710125, China; ^2^Department of Neurosurgery, Xijing Hospital, Fourth Military Medical University, Xi'an 710032, China; ^3^Department of Neurosurgery, Ankang Chinese Traditional Medicine, Ankang Hospital, Ankang 725000, China

## Abstract

Antiaging protein Klotho exhibits impressive properties of anti-inflammation, however is declined early after intervertebral disc injury, making Klotho restoration an attractive strategy of treating intervertebral disc inflammatory disorders. Here, we have found that Klotho is enriched in nucleus pulposus (NP) cells and Klotho overexpression attenuates H_2_O_2_-induced acute inflammation essentially via suppressing Toll-like receptor 4 (TLR4). The proinflammatory NF-*κ*B signaling and cytokine expressions paralleled with Klotho repression and TLR4 elevation in both NP cells (H_2_O_2_ treatment) and rat intervertebral disc (needle puncture treatment). Overexpression of TLR4 downregulated expression of Klotho, whereas interfering TLR4 expression diminished the inhibitory effects of H_2_O_2_ on Klotho in NP cells. Consistently, Klotho knockdown by RNA interferences largely diminished the anti-inflammatory and intervertebral disc protective effects in an Intervertebral Disc Degeneration (IDD) model. Thus, our study indicates that TLR4-NF-*κ*B signaling and Klotho form a negative-feedback loop in NP cells. Also, we demonstrate that the expression of Klotho is regulated by the balance between upregulation and downregulation of TLR4-NF-*κ*B signaling.

## 1. Introduction

Degeneration of the intervertebral disc is the main cause of lumbar protrusion. The lumbar intervertebral disc is the heaviest part of the human body. It is generally believed that the intervertebral disc begins to degenerate after the age of 20, but now, it is further proved that the degeneration started to occur at the age of 15. The water content of the nucleus gradually declines. Also, the elasticity and load-resistance of intervertebral disc lessen. Recent studies found that a series of inflammatory factors involved in the IDD process, which closely related to the occurrence and development of IDD [1–3]. However, the role of various inflammatory factors and the molecular mechanisms involved in IDD is not fully definitude.

The intervertebral disc consists of a central nucleus pulposus, an outer fibrous annulus, and an endplate at the upper and lower ends. Its main function is to maintain the normal spine structure and bear the biological stress of the spine. The NP tissue contains a large amount of water and proteoglycan, which is a necessary prerequisite for the physiological function and stress of the intervertebral disc. During embryogenesis of intervertebral disc cells, NP cells play a key role in initiating tissue formation and may be directly responsible for the development of the nucleus pulposus. In some species, like humans, NP cells may eventually be lost and replaced by chondrocyte-like cells until the disc is completely composed of fibers [4, 5]. Therefore, aging is one of the risk factors for the degeneration of intervertebral discs.

Klotho is a newly discovered antiaging gene. Loss of Klotho can cause multiple senescence-like phenotypes in mammals [6, 7]. In contrast, overexpression of Klotho in Klotho -/- mice prolongs their lifespan [8]. Klotho is a kidney-rich protein that plays a key role in mineral metabolism and kidney protection [7, 9–11]. It is also expressed in the heart, brain, and parathyroid [12–14]. The Klotho gene encodes a single transmembrane protein and secreted protein, which facilitates regulation of various cellular processes by affecting multiple cell membrane receptors and transporters and related signaling pathways such as aging, inflammation, apoptosis, oxidative stress, etc. Human Klotho gene polymorphisms are associated with pathophysiological bone loss in aging [15], spinal disease [16], osteocalcin levels [15], and bone mineral density [17]. Furthermore, the expression of Klotho protein in the intervertebral disc has been reported. However, its molecular mechanism of specific action in the intervertebral disc is still unclear.

Inflammatory factors are a class of substances involved in inflammatory reactions produced by cells and body fluids, which play a major role in the degeneration of intervertebral discs. They participate in and promote the progression of IDD [18]. A study have showed that the expression level of inflammatory factors in degenerated intervertebral disc tissue was significantly higher than that in the normal intervertebral disc tissue, and the expression level of inflammatory factors was positively correlated with the degree of degeneration of intervertebral disc [19]. Interleukin-1*β* (IL-1*β*), tumor necrosis factor *α* (TNF*α*), prostaglandin E2 (PGE2), and other inflammatory factors can be detected in degenerated intervertebral disc tissue [20, 21]. These inflammatory factors are the main factors leading to the inflammatory reaction of intervertebral disc tissue and played an important role in the process of IDD [22]. Therefore, the purpose of this study is twofold: to determine Klotho expression in nucleus pulposus cells and to determine the relationship between Klotho expression and inflammatory response during intervertebral disc degeneration. These studies will provide new insights into the treatment of IDD with Klotho-targeted inflammatory factors.

## 2. Materials and Methods

### 2.1. Animal Studies of IDD

All animals and the experimental procedures were performed with approval from the University Guidelines and approved by the Institutional Animal Care Committee (IACUC) of Xijing Hospital (2018007). Sprague-Dawley male rats of 12 weeks of age were purchased from the model animal research center of Xi Jing hospital and housed in the specific pathogen-free (SPF) conditions with the standard temperature (22 ± 1°C), humidity (50–60%), and light conditions (12 h light/dark cycle). Rat model of IDD was adopted from a previous study [23]. Rats were assigned to one of two groups (*n* = 6 in each group): (1) sham group: no treatment; (2) IDD group: needle puncture. For siRNA in vivo study, small interfering RNA (siRNA) of rat Klotho was employed, which targeted ATATT TATTG TAGAA AATGG. The control siRNA contained a scrambled RNA sequence. A single dose of siRNA (10 nm in 200 *μ*l of PBS) was applied to each rat through tail intravenous injection 1 day before IDD operation and then subjected to the IDD model twice a week before and during IDD (*n* = 6 in each group). After experiment completion, rats were sacrificed by CO_2_ inhalation, and rat caudal disc was surgically removed and stored at -80°C for further analysis.

### 2.2. Isolation of NP Cells

In total, 20 12-week-old Sprague-Dawley male rats were used for this study. NP cells were isolated by using reported methods [24]. In brief, the rats were euthanized by injection of an overdose of sodium pentobarbital. The caudal disc was separated under aseptic conditions. The gelatinous NP tissue was completely removed by microscopic sputum and placed in a petri dish containing PBS. The tissue were cut into 1 mm × 1 mm × 1 mm blocks by ophthalmic scissors, and the tissue suspension was collected and centrifuged at 1000 rpm for 5 min. The pellet were placed in 2 volumes of 0.1% type II collagen and shaken at 37°C for 4 h, then collected the cell suspension, centrifuged at 1000 rpm for 5 min, discarded the supernatant, and finally resuspended in complete medium (containing 10% FBS, 1% penicillin-streptomycin in DMEM/F12 medium). The cells were counted, inoculated in a 25 cm^2^ flask, and cultured in a saturated humidity incubator (37°C, 5% CO_2_). After 4 days, the liquid was changed for the first time. The cells were passaged for 90% fusion and inoculated into a new culture flask at a ratio of 1 : 2. The time of adherence and the time of cell growth to 90% fusion were recorded separately. Because cells obtained from the rat intervertebral disc tissues were variable in morphology until passage 2 to 3, we used passage (<3) cells cultured in monolayers for all experiments.

### 2.3. Immunofluorescence Staining

After NP cells were cultured on cover slips and treated, 4% PFA (paraformaldehyde) fixed at room temperature for 30 min, 1 × PBS washed 3 times for 5 min each time, 0.1% Triton X-100 (diluted with 1 × PBS) transparented for 10 min, 1 × PBS washed 3 times for 5 min each time, 5% BSA blocked at 37°C for 1 h, primary antibody (diluted with 5% BSA) incubated at 4°C overnight, 1 × PBST washed 3 times for 5 min each time, secondary antibody (diluted with 5% BSA) incubated at 37°C for 1 h free to light, 1 × PBST washed 5 times for 5 min each time, DAPI (1 *μ*g/ml) incubated 5 min, and 1 × PBST washed 5 times for 5 min each time. Finally, images were collected by confocal microscopy (Olympus, Japan).

### 2.4. Histology Study

Intervertebral disc sections were prepared, and the sections (4 *μ*m) were stained by haematoxylin and eosin (H&E) method. The histological samples were selected blindly by a lab observer. Normal discs showed well-organized collagen lamelas in AF and evenly distributed NP cells. After injury, the size of the NP decreased with the nuclear cells clustered and separated by dense areas of proteoglycan matrix, and the collagen layers became disorganized. Ten randomly selected fields were examined for each animal.

### 2.5. Reverse-Transcription PCR (RT-PCR) and Quantitative Real-Time PCR (qRT-PCR)

Total RNA was extracted from rat nucleus pulposus cells or tissues by using the TRIzol RNA isolation protocol (Invitrogen). The cDNA was synthesized by using a FastKing RT kit (with gDNase) (Qiagen). RT-PCR and qRT-PCR were performed by using a SuperReal PreMix Plus kit (SYBR Green) (Qiagen). All primers were synthesized by Takara Bio, Inc. (Tokyo, Japan). IL-1*β*: F-CACCT CTCAA GCAGA GCACA G, R-GGGTT CCATG GTGAA GTCAA C; NOS2: F-GACCA GAAAC TGTCT CACCT G, R-CGAAC ATCGA ACGTC TCACA; IL-18: F-ATATC GACCG AACAG CCAAC, R-TTCCA TCCTT CACAG ATAGG G; GAPDH: F-GGCAC AGTCA AGGCT GAGAA TG, R-ATGGT GGTGA AGACG CCAGT A.

### 2.6. Plasmid Constructions, Overexpression, and Knockdown

HA-tagged mouse Klotho (pUSEHA) and myc-tagged TLR4 (pCDNA3.1) expression plasmids were constructed from nucleus pulposus cell mRNA by RT-PCR. The primer sets used were cKlotho-F: TAAGC GGCCG CCATG CCAGC CCGCG CCCCT, and cKlotho-R: CGCTC GAGTT ATTTA TAACG TCTCC GGCCT; cTLR4-F: CGGGA TCCAT GATGC CTCTC TTGCA TCTGG and cTLR4-R: GGGGT ACCTC AGGTC AAAGT TGTTG CTTCT (the restriction enzyme sites were underlined). The primer sets for shRNA-TLR4 were used for constructing shRNA plasmids at the BamHI and HindIII sites of GV102 vector. The primer sequences were as follows: F-GATCC CGGCA TAGAG GTACT TCCTA ATATT CAAGA GATAT TAGGA AGTAC CTCTA TGCTT TTTTG GAAA; R-AGCTT TTCCT AAAAA AGCAT AGAGG TACTT CCTAA TATCT CTTGA ATATT AGGAA GTACC TCTAT GCCGG. All primers were synthesized by Takara Bio, Inc. (Tokyo, Japan).

### 2.7. Western Blotting Analysis

#### 2.7.1. Extraction of Total Protein from Cells and Tissue

In brief, add 1 ml of lysate plus 10 *μ*l of PMSF (100 mM) to cells and tissue, after lysing for 30 min; the lysate can be transferred to a 1.5 ml centrifuge tube with a pipette, then centrifuged at 12000 rpm for 5 min at 4°C, and the supernatant is placed in a 0.5 ml centrifuge tube and placed at -20°C. 30 to 60 *μ*g of protein/lane was subjected to electrophoresis by using 10% sodium dodecyl sulfate-polyacrylamide gel electrophoresis (SDS-PAGE) (Bio-Rad, Hercules, CA, USA). The resolved proteins were transferred electrophoretically to nitrocellulose membrane “blots.” The blots were blocked with 5% BSA in TBST (50 mM Tris, pH 7.6, 150 mM NaCl, 0.1% Tween 20) and incubated overnight at 4°C in 5% BSA in TBST with anti-Klotho (1 : 1,000; Cell Signaling), anti-TLR4 (1 : 1,000; Abcam), and anti-NF-KB (1 : 1,000; Cell Signaling). Immunolabeling was detected with electrochemiluminescence reagent (Amersham Biosciences, Piscataway, NJ, USA). Quantification of Western blots was performed by using ImageJ pixel analysis (NIH Image software). Data from Western blots are presented as band density normalized to the loading control (actin).

### 2.8. Transfections

In brief, prepare the cells: inoculated with 0.5 − 2 × 10^5^ nucleus pulposus cells in 500 *μ*l of complete medium without antidouble antibody 24 h before transfection, the cell fusion degree was 80-90% when transfected (note: when planting the plate, thoroughly digest the cells to avoid cell accumulation). For each transfection sample, prepare the following: dilute 0.8 *μ*g of plasmid DNA with 50 *μ*l Opti-MEM, gently pipette for 3-5 times, and let stand for 5 min at room temperature; mix the transfection reagent by gently inverting, dilute 2.0 *μ*l of Lipofectamine 2000 (Invitrogen) with 50 *μ*l Opti-MEM, gently aspirate for 3-5 times, and let stand for 5 min at room temperature; mix the transfection reagent and the plasmid DNA dilution, mix gently for 3-5 times, and let stand for 20 min at room temperature (note: once the transfection complex is formed, it should be immediately added to the culture dish for cell transfection); the transfection complex was added to a 24-well cell plate at 100 *μ*l/well, and the cell plate was gently shaken before and after; place the cell plate at 37°C in a 5% CO_2_ incubator for about 6 h, change the medium, replace it with ordinary medium containing 10% serum, and continue to culture for 24 h at 37°C in a 5% CO_2_ incubator. To check the transfection efficiency, nucleus pulposus cells were transfected with plasmid encoding green fluorescent protein; the transfection efficiency for nucleus pulposus cells was 60% to 70%. In addition, the overexpression or silencing efficiency of the targets was confirmed at the mRNA level or protein level.

### 2.9. Statistical Analysis

Typically, data were compiled from at least three independent triplicate experiments, each performed on separate cultures and on separate occasions. Data are presented as mean ± standard deviation (SD). Comparisons of data between groups were performed by using the Student *t* test or ANOVA for assessing variance. *P* < 0.05 or *P* < 0.01 was considered statistically significant or very significant.

## 3. Results

### 3.1. The Expression of Decreased Klotho and the Activation of TLR4-NF-*κ*B Signaling by Degenerative Intervertebral Disc

**Figure 1:**
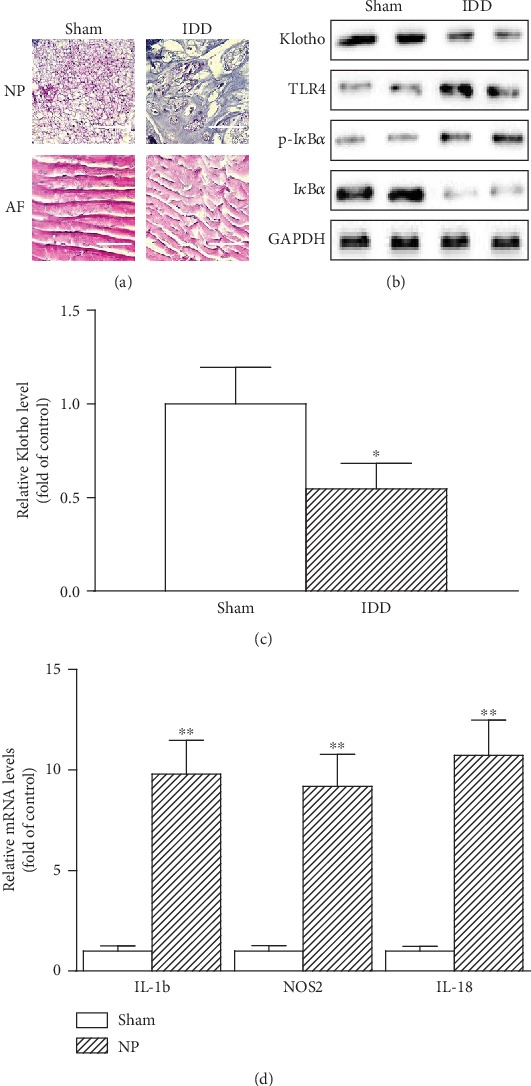
The expression of Klotho decreased and the activation of TLR4-NF-*κ*B signaling in degenerative intervertebral disc. Rats were treated with or without needle puncture (np) for 2 weeks. (a) Representative photomicrographs of H&E-stained intervertebral disc sections (*n* = 6 in each group). Typical histologic appearance of the annulus fibrosus (AF) and NP from normal and degenerated discs is induced by percutaneous needle puncture. Scale bar, 20 *μ*m (original magnification, ×40). (b) Representative protein levels of Klotho, TLR4, p-I*κ*B*α*, and I*κ*B*α* assayed by Western blotting (two randomly selected samples from each group were shown). (c) Klotho mRNA levels by qPCR. (d) Average mRNA levels of IL-1*β*, NOS2, and IL-18 were assayed by qRT-PCR and quantified as fold changes (*n* = 6 in each group). Data were presented as mean ± SD. ^∗^*P* < 0.05, ^∗∗^*P* < 0.01 vs. sham.

To gain insight into the connection of Klotho and TLR4-NF-*κ*B signaling in degenerative intervertebral disc, we adopted a rat model of IDD. Rat receiving needle puncture for 2 weeks expectedly displayed severe intervertebral disc damage. Normal discs showed well-organized collagen lamelas in AF and evenly distributed NP cells. After injury, the size of the NP decreased with the nuclear cells clustered and separated by dense areas of proteoglycan matrix, and the collagen layers became disorganized (Figure 1(a)). The expression of both the Klotho protein and the mRNA decreased (Figures 1(b) and 1(c)). However, the expression of TLR4 and p-I*κ*B*α* is elevated, accompanied by I*κ*B*α* protein reduction and hyperinflammatory cytokine inductions of IL-1*β*, NOS2, and IL-18 (Figure 1(d)). These indicated that the expression of Klotho decreased and the activation of TLR4-NF-*κ*B signaling in degenerative intervertebral disc.

### 3.2. Decreased Klotho Expression in NP Cells Treated with H_2_O_2_

**Figure 2:**
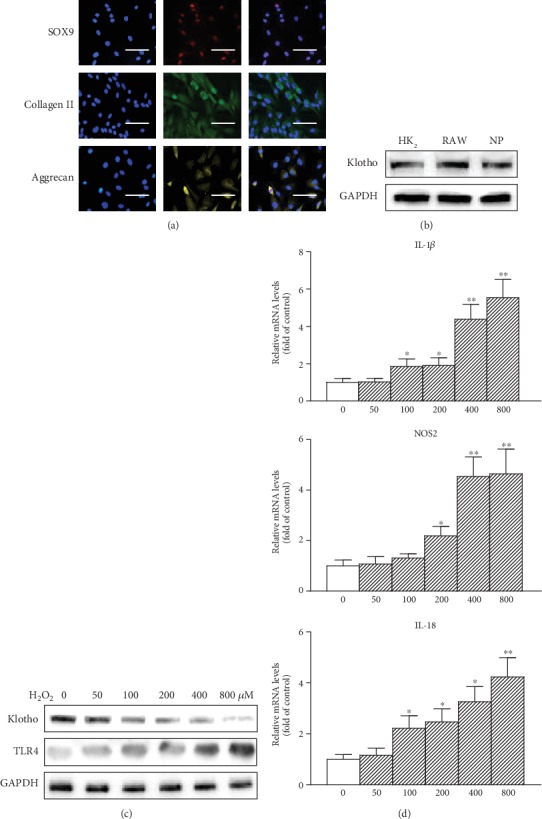
Decreased Klotho expression in NP cells treated with H_2_O_2_. (a) Primary NP cells were stained by SOX9, collagen II, and Aggrecan. To confirm the purity of in vitro primary NP cells culture, one representative experiment of six was shown. Scale bar, 20 *μ*m (original magnification, ×40). (b) Klotho protein expression was tested from human renal tubule epithelium HK2 cells, mouse macrophage RAW264.7 (RAW), and NP cells by Western blotting. (c) NP cells were treated with increasing amounts of H_2_O_2_ (0, 50, 100, 200, 400, and 800 *μ*M) for 12 h, and then Klotho and TLR4 protein levels were assayed by Western blotting. (d) NP cells were treated with increasing amounts of H_2_O_2_ (0, 50, 100, 200, 400, and 800 *μ*M) for 6 h, and then IL-1*β*, NOS2, and IL-18 mRNAs were assayed by qRT-PCR. GAPDH served as an internal control. The results were presented as mean ± SD of three independently performed experiments. ^∗^*P* < 0.05, ^∗∗^*P* < 0.01 vs. control.

The primary NP cells was collected and stained by SOX9, collagen II, and Aggrecan, which confirmed that more than 99% of these cells were NP cells (Figure 2(a)). Klotho is reportedly expressed in the kidney, mouse macrophage RAW264.7 (RAW) [25]. We detected Klotho protein in rat NP cells (Figure 2(b)), which means that Klotho might directly regulate intervertebral disc local and systemic inflammation processes. NP cells expressed Klotho protein and mRNA, whereas H_2_O_2_ treatment dose-dependently decreased its abundance accompanied by a marked TLR4 protein elevation (Figure 2(c)). Furthermore, H_2_O_2_ dose-dependently raised the expression of IL-1*β*, NOS2, and IL-18 (Figure 2(d)), indicating that the H_2_O_2_ downregulation of Klotho likely relates to its elevation of TLR4 and inflammatory cytokine expression in NP cells.

### 3.3. Regulation of Klotho by TLR4-NF-*κ*B Signaling in NP Cells

**Figure 3:**
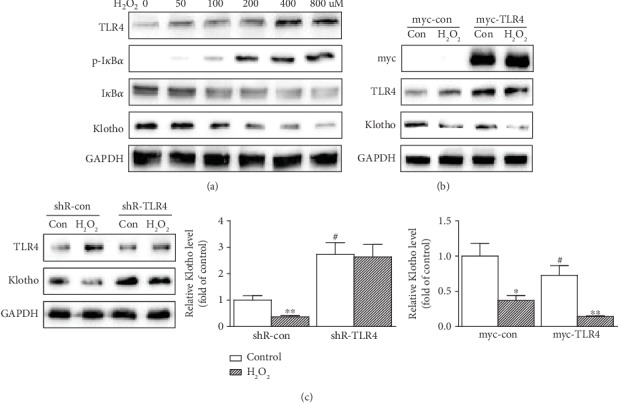
Regulation of Klotho by TLR4-NF-*κ*B signaling in NP cells. (a) NP cells were treated with increasing amounts of H_2_O_2_ (0, 50, 100, 200, 400, and 800 *μ*M) for 12 h, and then TLR4, p-I*κ*B*α*, I*κ*B*α*, and Klotho protein levels were assayed by Western blotting. (b) NP cells were transfected with control or a plasmid expressing myc-tagged TLR4 then treated with H_2_O_2_ (200 *μ*M) for 12 h. The overexpression of myc-tagged TLR4 was verified with an anti-myc antibody. TLR4 and Klotho were assayed by Western blotting. Bar graph on right: quantifications of Figure 3(b). (c) NP cells were transfected with a control or a shRNA-TLR4 plasmid then treated with H_2_O_2_ (200 *μ*M) for 12 h. Cell lysates were tested for TLR4 and Klotho expressions by Western blotting. Bar graph on right: quantification of Figure 3(c). GAPDH served as an internal control. The results were presented as mean ± SD of three independently performed experiments. ^∗^*P* < 0.05, ^∗∗^*P* < 0.01 vs. control. ^#^*P* < 0.05 vs. shR-con or myc-con.

In order to explore the potential correction of Klotho and TLR4-NF-*κ*B signaling in NP cells, we assessed whether TLR4 gain or loss affects the Klotho expression. The results showed that H_2_O_2_ induced TLR4 accumulation and TLR4-NF-*κ*B signaling activation, but the expression of Klotho blunted in NP cells (Figure 3(a)). Overexpression of myc-tagged TLR4 downregulated Klotho expression in NP cells (Figure 3(b)). On the contrary, the NP cells transfected with a small hairpin RNA (shRNA) specific for TLR4 diminished the inhibitory effects on Klotho (Figure 3(c)). These revealed that Klotho is regulated by TLR4-NF-*κ*B signaling in NP cells.

### 3.4. Klotho Suppression of the TLR4-NF-*κ*B Signaling in NP Cells

**Figure 4:**
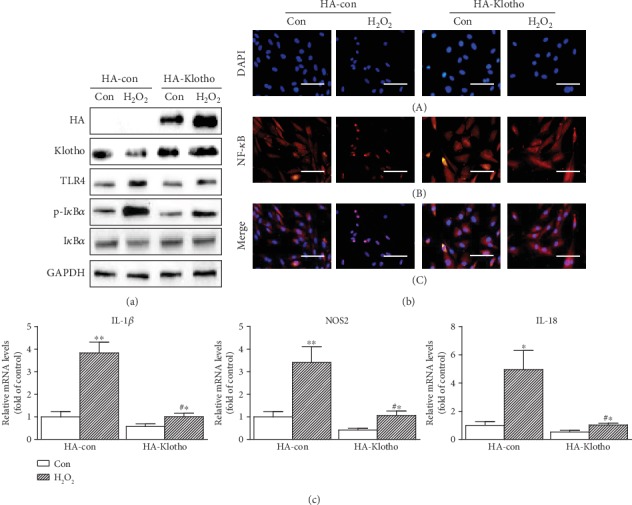
Klotho suppression of the TLR4-NF-*κ*B signaling in NP cells. (a) NP cells were transfected with HA-con and HA-Klotho plasmid for 24 h, then treated with H_2_O_2_ (200 *μ*M) for 12 h. The overexpression of HA-tagged Klotho was verified with an anti-HA antibody. Cell lysates were tested for Klotho, TLR4, p-I*κ*B*α*, and I*κ*B*α* expressions by Western blotting. (b) Immunofluorescent staining of NF-*κ*B nuclear translocation. NP cells transfected and treated as in Figure 4(a) for 30 min were stained with anti-NF-*κ*B antibody (B), counterstained with DAPI (A), and the figures were merged (C). Scale bar, 20 *μ*m (original magnification ×40). (c) NP cells transfected and treated as in Figure 4(a) for 6 h were tested for IL-1*β*, NOS2, and IL-18 mRNA levels by qRT-PCR. GAPDH served as an internal control. The results were presented as mean ± SD of three independently performed experiments. ^∗^*P* < 0.05, ^∗∗^*P* < 0.01 vs. control. ^#^*P* < 0.05 vs. H_2_O_2_.

To confirm the critical role of Klotho in the inhibition of TLR4-NF-*κ*B inflammation signaling, the effect of Klotho overexpression on inhibition of TLR4-NF-*κ*B signaling and inflammatory cytokine expressions was studied. Klotho overexpression in NP cells eliminated H_2_O_2_-induced TLR4 accumulation and I*κ*B*α* degradation (Figure 4(a)) and abrogated NF-*κ*B nuclear translocation (Figure 4(b)). H_2_O_2_-induced proinflammatory cytokine expression of IL-1*β*, NOS2, and IL-18 was also significantly abolished in cells overexpressing Klotho (Figure 4(c)), indicating that Klotho effectively suppressed the TLR4-NF-*κ*B signaling in NP cells.

### 3.5. Functional Role of Klotho in Degenerative Intervertebral Disc

**Figure 5:**
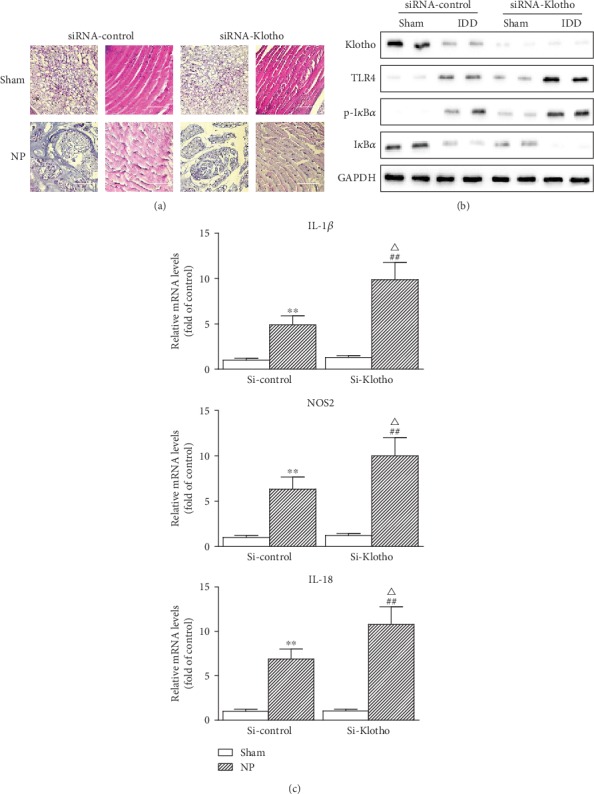
Functional role of Klotho in degenerative intervertebral disc. Rats injected with either siRNA-control or siRNA Klotho were treated with or without needle puncture for 2 weeks. (a) Representative photomicrographs of H&E-stained intervertebral disc sections (*n* = 6 in each group). Typical histologic appearance of the AF and NP from normal and degenerated discs induced by percutaneous needle puncture. Scale bar, 20 *μ*m (original magnification ×40). (b) Expressions of Klotho, TLR4, p-I*κ*B*α*, and I*κ*B*α* were assayed by Western blotting (two randomly selected samples from each group were shown). (c) mRNA levels of IL-1*β*, NOS2, and IL-18 from rats were assayed by qRT-PCR and quantified as fold changes (*n* = 6 in each group). Data were presented as mean ± SD. ^∗∗^*P* < 0.01 vs. sham in siR-control group; ^##^*P* < 0.01 vs. sham in siRNA Klotho group; ^△^*P* < 0.05 vs. IDD in siRNA-control group.

To further consider the in vivo relevance of Klotho, the effects of Klotho knockdown by small interfering RNA (siRNA) on anti-inflammation and intervertebral disc protection in a rat model of IDD were tested as shown in Figure 5. We injected rat with either siRNA-control or siRNA-Klotho, subjected rat to NP for 2 weeks, and then examined the intervertebral disc pathophysiological changes and proinflammatory cytokine expressions. Klotho knockdown noticeably caused the increase of intervertebral disc injuries, judged by AF and NP cells (Figure 5(a)), TLR4 and p-I*κ*B*α* accumulation, I*κ*B*α* reductions (Figure 5(b)), and enhanced expressions of IL-1*β*, NOS2, and IL-18 (Figure 5(c)). Thus, Klotho restoration not only inhibits TLR4 signaling and the associated inflammation in cells but also attenuates intervertebral disc injuries.

## 4. Discussion

**Figure 6:**
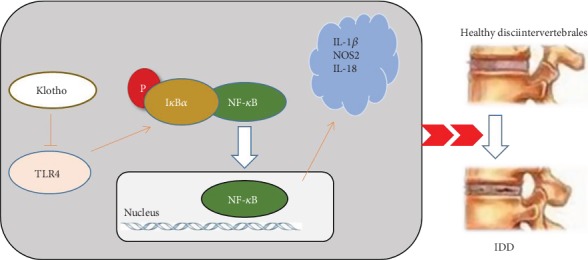
Schematic of Klotho suppression of TLR4 and inhibition of TLR4-NF-*κ*B inflammatory responses.

In this study, we have presented a novel finding in this study: TLR4-NF-*κ*B signaling and Klotho form a negative-feedback loop in NP cells, and the expression of Klotho is regulated by the balance between upregulation and downregulation of TLR4-NF-*κ*B signaling (Figure 6). Therefore, our results elaborate a novel mode of action through which Klotho converge on its anti-inflammation activities that might contribute significantly to its intervertebral disc and extrarenal protective functions.

We focused on the effects of expression changes of Klotho protein during intervertebral disc degeneration to elucidate the molecular mechanisms of intervertebral disc degeneration. Some studies have shown that Klotho expresses in the heart, aorta, kidney, brain, lung, liver, pancreas, spleen, skeletal muscle, and adipose tissue [26–28]. However, a study has demonstrated that Klotho protein is expressed in intervertebral disc [29].

The pathophysiological mechanism of IDD is still unclear. In recent years, tremendous efforts have been made to the investigation of pathophysiological mechanisms of IDD. It believes that the pathophysiological mechanisms mainly includes the following pathophysiological processes: decreasing synthesis of extracellular matrix, increasing secretion of enzymes that decompose extracellular matrix, increasing cell senescence and apoptosis, and invading intervertebral disc tissue by nerves and blood vessels [1]. Inflammatory factors are closely related to these processes. Previous studies have shown that Klotho inhibits the upregulation of TNF*α*-induced intercellular adhesion molecules and vascular cell adhesion molecules and the activation of NF-*κ*B, which directly resist the inflammatory effects of proinflammatory cytokines [30]. In addition, Liu et al. found that intracellular Klotho interacts with retinoic acid-inducible gene 1 in senescent cells and inhibits retinoic acid-induced gene-1-induced expression of interleukin 6 (IL-6) and IL-8, which confirmed that Klotho inhibits aging-related inflammatory responses [31]. After that, we studied the relationship between inflammatory signaling and Klotho. Finally, we studied the protective effects of Klotho in IDD.

It is clear that the degeneration of the intervertebral disc increases with age. We hypothesized that Klotho protein maintains NP homeostasis in normal intervertebral discs. Unexpectedly, we found that a decrease in Klotho expression in NP cells was caused by the activation of inflammatory signaling during degeneration of the intervertebral disc. As known, the Klotho protein consists of three parts: the extracellular domain, the transmembrane domain, and the intracellular domain. Membrane Klotho acts as a receptor that regulates phosphate excretion in the kidney and synthesis of active vitamin D [32, 33]. Secreted Klotho regulates the activity of multiple growth factors 11 [34]. Doi et al. reported that secreted Klotho inhibits TGF-*β*1 signaling by directly binding to the type II TGF-beta receptor (TGFbR2) on the cell surface and preventing TGF-*β*1 from binding to the receptor [35]. TGF-*β*1 is the most effective inducer of matrix synthesis in NP cells. Kuro-o found that Klotho can directly regulate the endocrine FGF family [36]. Therefore, the role of Klotho in nucleus pulposus cells may have many different outcomes. Future studies will focus on the human sample to determine the role of Wnt signaling to determine whether Klotho's regulation of cell growth and matrix synthesis is specific for NP cells. In addition, it is necessary to examine whether Klotho affects the activity of multiple signaling pathways in NP cells, including the activity of TGF, FGF, or MMP families that may be involved in disc degeneration.

## 5. Conclusions

We studied the expression of Klotho in the intervertebral disc and NP cells and elucidated the signal crosstalk between Klotho and the inflammatory signal, which is a potential trigger for disc degeneration. We demonstrated that the mutual antagonism of Klotho and TLR4-NF-*κ*B inflammatory pathways was discovered by preparing a nucleus pulposus inflammatory model. These experiments strongly suggest that activation of inflammatory signaling inhibited Klotho protein expression in NP cells. Conversely, increasing Klotho expression inhibits inflammatory signaling in NP cells. These results may support that Klotho is not only an antagonist of TLR4-NF-*κ*B inflammatory signaling but also of TLR4-NF-*κ*B signaling and Klotho form a negative-feedback loop in NP cells. To elucidate the mechanism that causes degeneration of the intervertebral disc, further studies are needed to determine which molecules on various inflammatory signaling pathways interact with Klotho to regulate nucleus pulposus cell growth and matrix synthesis.

## Data Availability

All data generated or analyzed during this study are included in this published article.
